# Total Knee Arthroplasty Violates the Law of Burmester—A Biomechanical Investigation

**DOI:** 10.3390/jpm13010036

**Published:** 2022-12-24

**Authors:** Günther Maderbacher, Armin Keshmiri, Hans Robert Springorum, Andreas Mösenbacher, Joachim Grifka, Clemens Baier

**Affiliations:** 1 Department of Orthopedic Surgery, University Medical Center Regensburg, 93053 Regensburg, Germany; 2 Department of Mechanical Engineering, University of Leoben, 8700 Leoben, Austria

**Keywords:** TKA, kinematics, total knee arthroplasty, Burmester, mid-flexion instability, knee

## Abstract

Background: Kinematic patterns of knees after total knee arthroplasty (TKA) are different from those of healthy knees. We hypothesised that these changes cause a relevant shift in the medial and lateral epicondyles and, consequently, the insertion sites of the collateral ligaments. Any alterations, however, violate the law of Burmester, which states a close relation between the course of the collateral and cruciate ligaments, and the articular surfaces. Methods: Ten healthy knees of whole body cadavers were investigated. The positions of the medial and lateral epicondyles in relation to the tibia were compared before and after cruciate retaining fixed bearing TKA between 0 and 90° of flexion using a navigational device. Results: After TKA, the medial and lateral epicondyles significantly shifted laterally (~3–5mm) between 0° and 40° of flexion. Additionally, the lateral epicondyle was located significantly more dorsal (~3–5mm) during 0° and 20° of flexion and significantly shifted proximally (~2.5–3mm) between 0° and 30° of flexion. Conclusions: By changing the epicondylar positions relative to the articular surfaces, the law of Burmester is violated in the present study setting. This might explain the impairment in motion, instability, or mid-flexion instability and the persistent pain in the knees after TKA.

## 1. Introduction

Kinematics of the knee consist of a complex combination of rotational and translational movement, often summarised as rolling and gliding [1]. The basic principle of motion can be represented by the mechanism of a four bar linkage showing a close relation between the function of the cruciate ligaments and shapes of the femoral condyles [2,3]. In the 1990s, the MRI technique provided new information regarding knee kinematics, proposing that the medial condyle almost does not translate but only rolls while the lateral condyle slides posteriorly during flexion [4].

**Figure 1:**
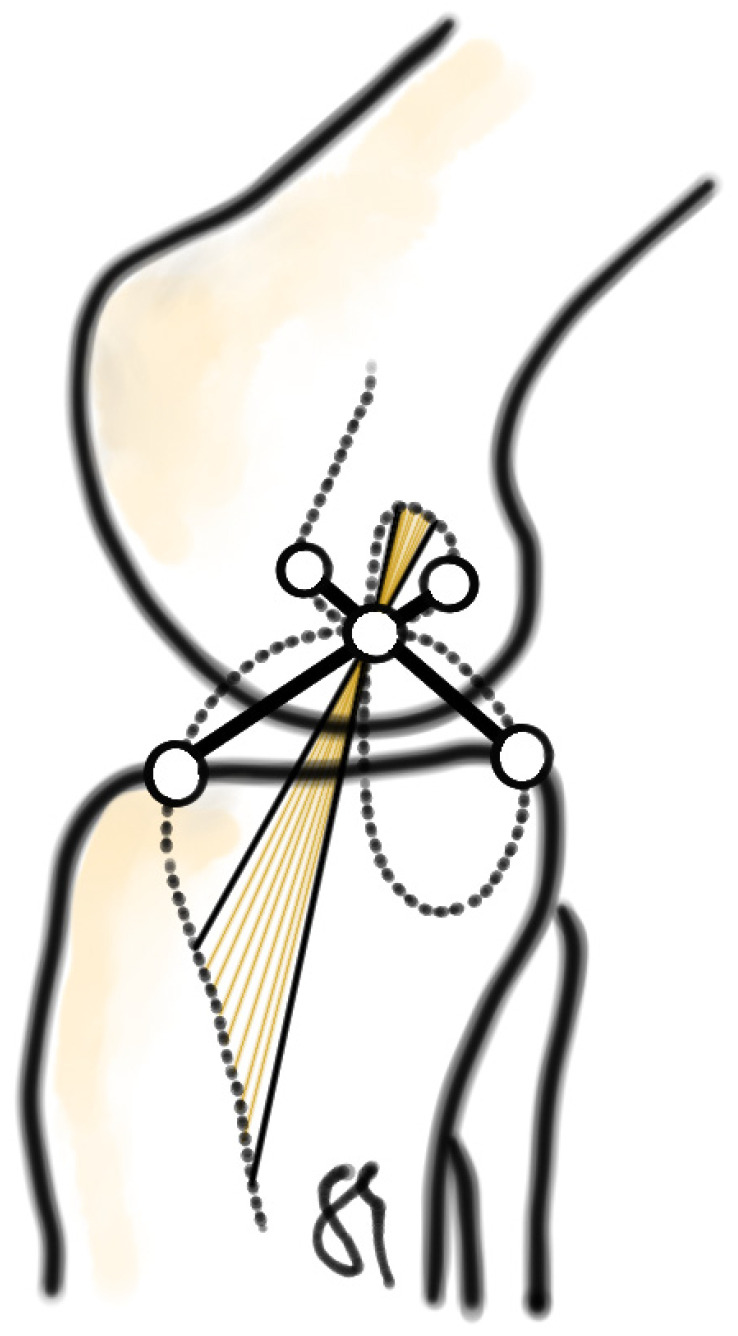
According to Burmester, fibres and insertion points of the collateral ligaments correspond to two-third-order curves, which are related to the articular surface and intersect at the crossing point of the four-bar linkage. This guarantees tensioned ligamentous fibres throughout flexion.

The insertion points and the structural anatomy of the medial and lateral collateral ligaments almost perfectly correspond to the Burmester curve, which consists of two-third-order curves, the “vertex-” and “pivot cubic” [2,5]. This mechanism guarantees tensioned ligamentous fibres with a defined laxity throughout flexion, which is necessary for the stability of the joint while still enabling the great extent of range of motion. Ideally, the cruciate and the collateral ligaments intersect at the crossing point of the four-bar linkage [2]. See Figure 1.

If, however, the origins of the collateral ligaments lie outside the ideal Burmester curve, the function of ligament fibres is impaired. They are either too long, resulting in laxity, or too short, blocking joint motion or being overstretched. Collagenous ligaments can be stretched by a maximum of 5% before being damaged [6].

Many recent studies revealed that joint kinematics and stability are altered after total knee arthroplasty (TKA) [7,8,9]. Moreover, osseous rotational and translational tibiofemoral parameters are significantly changed after TKA [10,11,12,13].

(1) We hypothesised that these changes in osseous rotational and translational alignment after TKA result in alterations in the positions of the medial and lateral epicondyles and, thus, in the origins of the medial and lateral collateral ligaments. Therefore, TKA violates the law of Burmester. If so, (2) we asked whether TKA causes a defined pattern of changes in epicondylar positions or if they occur randomly.

## 2. Material and Methods

For the present study, ten knees of Thiel embalmed whole cadavers without any history of surgeries on the lower limbs, osteoarthritis of the hips or knees, or fractures were investigated. Both for assessing knee kinematics and TKA, a commercially available CT-free navigation tool was utilised (Brainlab Knee 2.6, Brainlab, Feldkirchen, Germany) [10,11,12,13,14].

### 2.1. Surgical Procedure

After a median skin incision, the medial capsular structures were marked by a water proof pen. The joints were opened by a parapatellar medial arthrotomy. Great attention was paid to not harm any intracapsular structures such as the ligaments or menisci. Passive optical reference arrays were extra-articularly fixed to the femoral and tibial bones by Schanz screws. The femoral head centre was identified by circumduction. Using an optical pointer the required landmarks were digitised (femur: distal femoral knee centre, medial and lateral epicondyle, Whiteside line, and articulating surface of the medial and lateral condyle; tibia: tibial plateau magnitude, medial and lateral malleolus, Akagi line [15] defining the antero-posterior (AP) tibial axis, and the articulating surface of the medial and lateral tibial plateau). The capsule was anatomically closed according to the markings. Using a continuous passive motion (CPM) device, the knees performed a motion with 0° and 90° flexion. During two full motion cycles, the kinematics of the knees/lower limbs were recorded by the navigation device.

Thereafter, navigation-based TKA (DePuy PFC Sigma cruciate retaining with a fixed bearing inlay, DePuy, Warsaw, IN, USA) was performed. The menisci and the anterior cruciate ligament were removed. The tibial cut was made perpendicular to the mechanical axis with 4° of slope. From the healthy lateral tibial compartment, 8 mm of the bone/cartilage was removed. A double tensiometer with a metric scale (Knee Balancer, DePuy, Warsaw, IN, USA) was inserted with a distraction force of 90 N to assess the gaps in extension and 90° of flexion. The distal femoral cut was made perpendicular to the femoral mechanical axis. The flexion gap was equally balanced by adjusting femoral rotation and femoral component size. No ligamentous releases were performed. The tibial component was slightly externally rotated in relation to the Akagi line [15]. Regarding mediolateral alignment, both femoral and tibial components were orientated centrally, with the best coverage of the bone without overhang. To guarantee stable trials during motion, the components were fixed by screws and pins. After anatomical closure of the capsule, kinematics of the knees/lower limbs were recorded between 0° and 90° of flexion within two motion cycles. All surgeries were performed by one experienced surgeon (>500 navigated TKA).

### 2.2. Calculations

An anatomy-based coordinate system, defining a tibial and femoral matrix was used [16]. For the tibial matrix, the mechanical tibial axis (connecting line between the tibial plateau magnitude and the centre of the talus) was defined as *z*-axis ($z^{t}$). For the *y*-axis ($y^{t}$), the Akagi line [15] intersecting the *z*-axis was utilised. The *x*-axis ($x^{t}$) was calculated as the cross product of the two other axes. In the femoral matrix, the *z*-axis ($z^{f}$) also was defined as the mechanical femoral axis (connection between the femoral head centre and the distal femoral knee centre). A line connecting the medial and lateral epicondyle and intersecting the *z*-axis was defined as *x*-axis ($x^{f}$). The left *y*-axis ($y^{f}$) was calculated by the cross product of the first two axes. Motion between the femur and tibia is expressed by means of a third coordinate system ($x^{k},\;y^{k},\;z^{k}$) in which the anatomy based axes of the tibial and femoral coordinate system are utilised: internal and external tibial rotation is defined around an axis ($z^{k}$), which corresponds to the mechanical axis ($z^{t}$) of the tibia. Flexion and extension are defined around an axis ($x^{k}$), which corresponds to the femoral transepicondylar axis ($x^{f}$). Calculation of the cross product between the femoral mechanical axis ($z^{f}$) and the transepicondylar axis ($x^{f}$) determined a third floating axis ($y^{k}$) describing abduction and adduction. For joint translation, the same axes are used, while mediolateral shift occurs along the ($x^{k}$) axis, proximodistal shift along the ($z^{k}$) axis and ventrodorsal shift along the ($y^{k}$) axis. By means of a transformation matrix, the femoral matrix is transformed into the tibial matrix by building a homogenous matrix that combs 3 × 3 parameters for rotation (*a*, *b*, *c*, *e*, *f*, *g*, *i*, *j*, *k*) and three parameters for translation (*d*, *h*, *l*).

$$\left[\begin{array}{c} \begin{array}{c} X^{\prime }\;\\ Y^{\prime }\\ Z^{\prime } \end{array}\\ \;0\; \end{array}\right]=\left[\begin{array}{c} \begin{array}{ccc} a & b & c\\ e & f & g\\ i & j & k\\ 0 & 0 & 0 \end{array} \end{array}\right]\;\left[\begin{array}{c} \begin{array}{c} d\;\\ h\\ l \end{array}\\ 1 \end{array}\right]$$

Relative positions of the medial and lateral epicondyles in relation to the tibia were calculated before and after TKA between 0° and 90° of flexion using the following formula.

$$X^{\prime }=\left(X\times a\right)+\left(Y\times b\right)+\left(Z\times c\right)+\left(d\right)\;Y^{\prime }=\left(X\times e\right)+\left(Y\times f\right)+\left(Z\times g\right)+\left(h\right)\;Z^{\prime }=\left(X\times i\right)+\left(Y\times j\right)+\left(Z\times k\right)+\left(l\right)$$

Positive values were defined as lateral, dorsal, and distal shifts in a condyle.

### 2.3. Statistical Methods

Absolute and relative differences of the positions of the medial and lateral epicondyles before and after TKA in three planes (x, y, and z) were calculated. Continuous variables are presented as mean value and standard deviation. To compare shifts (relative and absolute) before and after TKA, a paired t-test was used. A two-sided *p*-value of ≤0.05 was considered statistically significant. All analyses were conducted with IBM SPSS Statistics 21.0.0.

According to our local ethical committee, no approval was necessary. All methods were carried out in accordance with relevant guidelines and regulations.

## 3. Results

### 3.1. Relative Shift

**Figure 2:**
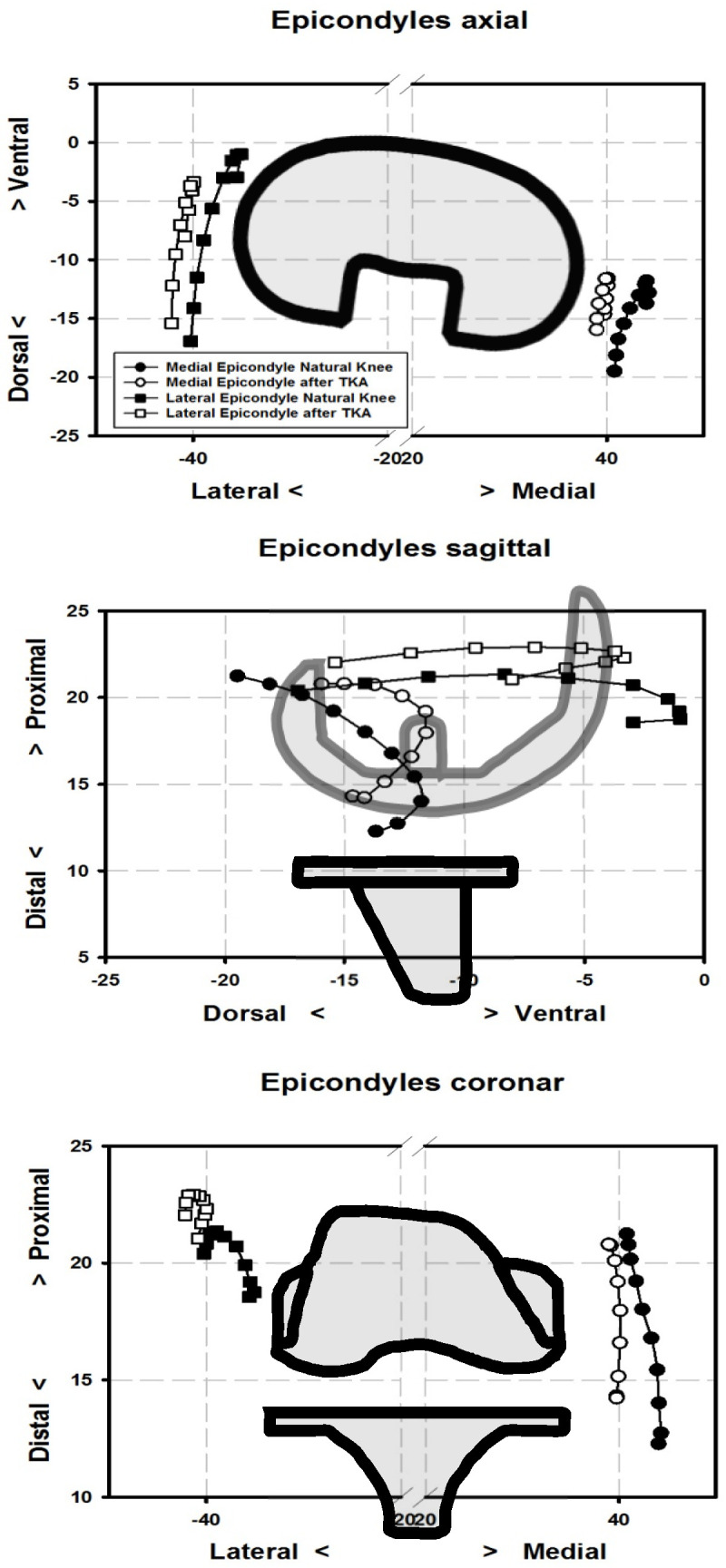
Positions of the medial epicondyles before (black dots) and after total knee arthroplasty (TKA) (white dots), and lateral epicondyles before (black squares) and after TKA (white squares) in millimetres between 0 and 90° of flexion are shown.

After TKA, medial epicondyles significantly shifted laterally between 0° and 40° of flexion (~3–5 mm). Lateral epicondyles also showed a significant lateral shift during the first 40 degrees of flexion (~3–5 mm). In contrast to medial epicondyles, lateral epicondyles were located significantly more dorsal during 0° and 20° of flexion (~3–5 mm) and significantly shifted proximally between 0° and 30° of flexion (~2–3 mm). See Table 1 and Table 2 and Figure 2.

### 3.2. Absolute Shift

After TKA, both the medial and lateral epicondyles showed significant absolute proximodistal, mediolateral and ventrodorsal shifts between 0° and 90° of flexion. See Table 3 and Table 4.

## 4. Discussion

The main finding is that, in the present study, the positions of both the medial and lateral epicondyles of the femoral bones in relation to the tibia were changed after total knee arthroplasty. These changes mainly occurred between 0° and 40° degrees of flexion: The medial and lateral epicondyles showed a significant lateral shift during the first 40° of flexion. The lateral epicondyles demonstrated an additional significant dorsal and proximal shift between 0 and 30° of flexion. As the insertion points of the collateral ligaments in relation to the articular surface were changed after TKA, Burmester’s law was violated. Thus, our hypothesis was confirmed.

The present results corresponded well with recent studies in which the translational and rotational changes between femoral and tibial bones, and the changes in the functional flexion axis after total knee arthroplasty are stated [10,11,12,13,17]. It has been shown that femoral bones in relation to the tibial bones are translated laterally after TKA. Incongruences between the proximal tibial bone anatomy and tibial implants, especially when slightly externally rotating some components, when considering symmetric components that do not respect the natural articulation points, and when applying surgical techniques such as the resection of the anterior cruciate ligament (ACL), were identified as possible reasons for that phenomenon [12]. In another study, a significant relation between the rotational alignment of both femoral and tibial components and the rotational alignment of the lower limb after TKA was found [10,13,18]: the external rotation of the femoral component resulted in the external rotation of the tibia or lower thigh, and vice versa, internal rotation of the lower thigh resulted when the femoral component was rotated internally. In contrast, external rotation of the tibial component caused internal rotation of the lower thigh, and internal rotation of the tibial component caused external rotation of the lower thigh. This was attributed to the conformity between tibial and femoral components in extension achieved by the large radii of the femoral condyles, which ensures the stability necessary during walking. This well corresponds to the data in the present study: In case of femoral external rotation or tibial internal rotation the lateral epicondyle shifts dorsally. Moreover, the lateral epicondyle showed a proximal translation near extension. This might be explained by the present implantation technique performing proximal tibial and distal femoral cuts perpendicular to the mechanical axes (mechanical alignment).

Besides these abovementioned significant directed shifts near extension (between 0° and 30–40° of flexion), absolute shifts (absolute values) show that there is a significant shift in both the medial and lateral epicondyles in the ventrodorsal, mediolateral and proximodistal directions throughout the rest of flexion as well (40–90°) (see Table 3 and Table 4). Although this implies relevant changes in the translational positions of the epicondyles, they occur randomly regarding directions and signs, respectively.

Which consequences arise from changed epicondylar positions? The medial collateral ligament is inserted at the superficial and deep layer around the medial epicondyle. The lateral collateral ligament is inserted around the lateral epicondyle. It has been shown that fibres of the medial and lateral collateral ligaments do not have a random course, but both are strictly related to the Burmester curve. Interestingly, the cruciate ligaments and the collateral ligaments all intersect at the crossing point of the four-bar linkage [2,3,5]. This guarantees taut ligaments throughout flexion, stabilising the knee in all directions. Besides the mentioned passive ligamentous stabilisation system, there also exist active stabilisation systems (vastus medialis, adductor, and semimembranosus and pes anserinus for the medial side and the popliteal muscle, the iliotibial tract, and the biceps muscle for the lateral side). Any alterations to the positions of the epicondyles or the distal femur in relation to the tibia change the insertion points of the medial and lateral collateral ligaments, the cruciate ligaments, and the active stabilisation system in relation to the joint surface. As form follows function in the knee, joint surfaces and positions of the ligaments are strictly related to each other. Any changes violate the law of Burmester by influencing a coherent and consistent system in the knee: it has been shown that shifting the ligament insertion of the medial collateral ligament anteriorly causes an theoretical elongation of the ligament of ~17% during flexion, resulting in damage to the ligament (collagenous ligaments can be stretched no more than 5% before being damaged [6]) or inhibiting further flexion [5]. As insertion points of the active stabilisation system are changed as well, their capability to actively guide and stabilise motion might be impaired and any tensioning might result in pain [19].

Mid-flexion instability is increasingly considered by orthopaedic surgeons in TKA. It is defined as valgus or varus instability between 30° and 60° of flexion. In the literature, this phenomenon is attributed to an elevated joint line, multi-radii design of the femoral component, and laxity of the collateral ligaments [20]. Relative changes in the insertions of the collateral ligaments, as shown in the present study, might contribute to ligamentous laxity being responsible for mid-flexion instability. This, however, needs to be investigated in further clinical and biomechanical studies.

The present study has several limitations. Unloaded knees were used for all investigations. This neglects the influence of muscles on knee kinematics. Pinskerova et al. [4] were able to show in MRI series that there are kinematical differences between loaded and unloaded knees. These differences were predictable and rather limited. In the present setting, cadavers fixed according to the Thiel method were used as they provide physiological soft tissue characters and excellent movement patterns [21,22]. Additionally, whole cadavers were investigated by assessing the influence of the entire surrounding soft tissues and muscles, the hip, as well as the muscles associated with the hip. In laboratory studies, knees are often cut on the level of femoral and tibial midshaft and forces of only some muscles are imitated by fixed weights put on clamps.

Regarding the registration process of the navigation system, it has been shown to be erroneous as well. However, the accuracy is described as an angular deviation of about less than 1° [14]. As maximum deviation was found for the kinematically assessed femoral head centre, only cadavers with healthy hips were investigated. Additionally, great attention has been paid to not moving the pelvis during circumduction to improve accuracy [14,23,24,25,26]. The main advantage of the present study setting is that only one test set-up/registration procedure is required to investigate kinematics of healthy knees and knees after TKA: the necessary registration procedure of bony landmarks just has to be performed once, whereas kinematic motion can be compared directly without running the risk of creating registration errors between implantations. Any wrong assessments of landmarks, therefore, cause a systematic error that does not mandatorily influence relative measurements, which were used for present calculations. Additionally, the navigation device enables a precise component alignment, again being related to the coordinate system that is used for kinematic assessment. Kinematics of the knees can also be assessed by fluoroscopic 3D-matching techniques, which advantageously allow for investigations under weight-bearing conditions [27,28]. However, patients are exposed to radiation, and real-time kinematic examinations throughout flexion are not possible. Kinematic changes have to be re-assessed in every single radiograph, which is prone to measurement errors. It must also be stated that, in general, a comparison of published data is difficult because of the different definitions of kinematics and movement that are used. This phenomenon is referred to as ‘kinematic crosstalk’ [24].

In the present study, only one implant and one surgical technique were used. The results might not be applicable to other implants and surgical techniques.

In future, we need further insights in order to evaluate the violation of Burmester’s law with other surgical techniques, i.e., kinematic alignment and taking into account the activity of the muscles. We also have to verify the corrective effects of knee prosthesis in pathological knees with varus/valgus deformity, in which Burmester’s law is probably already violated in pre-surgical phase.

## 5. Conclusions

In the present study, the positions of the medial and lateral epicondyles were changed after total knee arthroplasty. Both the medial and lateral epicondyles were shifted laterally in relation to the tibia between 0 and 40° of flexion. Additionally, the lateral condyle showed a significant dorsal and proximal shift during the first 30° of flexion. This might be the result of implant geometry neglecting natural anatomy as well as surgical technique. As a consequence, the law of Burmester, which shows a close relation between the course of collateral and cruciate ligaments and the articular surface, might be violated. This might be one reason for the impairment in motion and the instability of knees (mid-flexion instability) after TKA.

## Figures and Tables

**Table 1:** Relative shift in the medial epicondyle after total knee arthroplasty (TKA). Relative shifts in the medial epicondyle (mediolaterally, ventrodorsally and proximodistally) after TKA are shown in millimetres (means with standard deviations (SD)) every 10° of flexion. Positive values show a lateral, dorsal, and distal shift in the epicondyle. Bold characters mark significant differences (*p* < 0.05).

Flexion (°)	Mediolateral (mm)		*p*-Value	Ventrodorsal (mm)		*p*-Value	Proximodistal (mm)		*p*-Value
	Mean	SD		Mean	SD		Mean	SD	
0	4.3	3.6	**0.004**	0.9	8.2	0.727	−2.0	3.4	0.089
10	4.6	4.0	**0.006**	1.4	7.5	0.582	−1.5	3.3	0.188
20	4.2	4.1	**0.011**	1.5	6.3	0.461	−1.1	2.9	0.244
30	3.8	4.4	**0.022**	0.1	5.5	0.947	−1.2	2.4	0.156
40	3.2	4.2	**0.039**	−1.4	5.2	0.406	−1.2	1.8	0.063
50	2.5	4.0	0.076	−2.5	5.5	0.179	−1.2	1.4	**0.027**
60	2.2	3.7	0.093	−2.9	5.6	0.140	−0.9	1.4	0.078
70	2.0	3.4	0.092	−3.0	5.5	0.115	−0.6	1.5	0.257
80	2.0	3.5	0.098	−3.1	5.8	0.124	0.0	1.8	0.967
90	1.9	3.7	0.145	−3.5	6.0	0.092	0.4	2.1	0.524

**Table 2:** Relative shift in the lateral epicondyle after total knee arthroplasty (TKA). Relative shifts in the lateral epicondyle (mediolaterally, ventrodorsally, and proximodistally) after TKA are shown in millimetres (means with standard deviations (SD)) every 10° of flexion. Positive values show a lateral, dorsal, and distal shift in the epicondyle. Bold characters mark significant differences (*p* < 0.05).

Flexion (°)	Mediolateral (mm)		*p*-Value	Ventrodorsal (mm)		*p*-Value	Proximodistal (mm)		*p*-Value
	Mean	SD		Mean	SD		Mean	SD	
0	5.3	3.8	**0.002**	5.0	3.3	**0.001**	−2.5	3.4	**0.048**
10	5.4	4.1	**0.002**	4.8	3.0	**0.001**	−2.9	3.0	**0.014**
20	4.6	4.2	**0.008**	3.0	2.9	**0.009**	−2.9	2.8	**0.010**
30	4.0	4.5	**0.022**	1.8	3.0	0.087	−2.4	2.8	**0.022**
40	3.4	4.4	**0.037**	0.7	3.4	0.516	−2.0	2.8	0.052
50	2.7	4.1	0.069	−0.5	4.2	0.691	−1.7	3.0	0.099
60	2.4	3.8	0.074	−1.3	3.9	0.331	−1.6	2.9	0.126
70	2.2	3.3	0.069	−2.0	3.7	0.126	−1.7	2.8	0.090
80	2.2	3.3	0.070	−1.9	2.7	0.054	−1.8	2.6	0.059
90	1.9	3.6	0.124	−1.5	3.0	0.145	−1.6	2.7	0.085

**Table 3:** Absolute shift in the medial epicondyle after total knee arthroplasty (TKA). Absolute shifts in the medial epicondyle (mediolaterally, ventrodorsally, and proximodistally) after TKA are shown in millimetres (means with standard deviations (SD)) every 10° of flexion. Bold characters mark significant differences (*p* < 0.05).

Flexion (°)	Mediolateral (mm)		*p*-Value	Ventrodorsal (mm)		*p*-Value	Proximodistal (mm)		*p*-Value
	Mean	SD		Mean	SD		Mean	SD	
0	4.3	3.6	**0.004**	6.8	4.1	**0.001**	2.9	2.6	**0.006**
10	4.7	3.9	**0.004**	6.3	3.9	**0.001**	2.7	2.3	**0.006**
20	4.4	3.8	**0.005**	5.0	3.8	**0.002**	2.2	2.1	**0.009**
30	4.6	3.5	**0.003**	4.0	3.5	**0.005**	2.0	1.6	**0.004**
40	4.1	3.2	**0.003**	4.0	3.4	**0.005**	1.4	1.6	**0.021**
50	3.7	2.7	**0.002**	4.2	4.1	**0.010**	1.4	1.2	**0.004**
60	3.4	2.5	**0.002**	5.0	3.6	**0.002**	1.4	0.8	**0.000**
70	3.1	2.3	**0.002**	4.9	3.6	**0.002**	1.4	0.8	**0.000**
80	3.1	2.4	**0.003**	5.2	3.8	**0.002**	1.5	1.0	**0.001**
90	3.2	2.5	**0.003**	5.3	4.3	**0.003**	1.7	1.3	**0.003**

**Table 4:** Absolute shift in the lateral epicondyle after total knee arthroplasty (TKA). Absolute shifts in the lateral epicondyle (mediolaterally, ventrodorsally, and proximodistally) after TKA are shown in millimetres (means with standard deviations (SD)) every 10° of flexion. Bold characters mark significant differences (*p* < 0.05).

Flexion (°)	Mediolateral (mm)		*p*-Value	Ventrodorsal (mm)		*p*-Value	Proximodistal (mm)		*p*-Value
	Mean	SD		Mean	SD		Mean	SD	
0	5.3	3.8	**0.002**	5.0	3.3	**0.001**	3.6	2.2	**0.001**
10	5.4	4.1	**0.002**	4.8	2.8	**0.000**	3.3	2.6	**0.003**
20	4.8	3.9	**0.004**	3.4	2.4	**0.001**	3.1	2.4	**0.003**
30	4.7	3.7	**0.003**	3.1	1.5	**0.000**	3.0	2.1	**0.001**
40	4.3	3.3	**0.003**	2.7	2.0	**0.002**	2.9	1.6	**0.000**
50	3.8	2.8	**0.002**	2.7	3.2	**0.026**	2.9	1.7	**0.000**
60	3.6	2.4	**0.001**	3.0	2.7	**0.007**	2.8	1.7	**0.001**
70	3.2	2.2	**0.001**	3.1	2.7	**0.005**	2.7	1.7	**0.001**
80	3.2	2.1	**0.001**	2.7	1.9	**0.002**	2.4	1.9	**0.004**
90	3.4	2.1	**0.001**	2.7	2.0	**0.002**	2.4	2.0	**0.005**

## Data Availability

The data presented in this study are available on request from the corresponding author.
